# The Gravity of Objects: How Affectively Organized Generative Models Influence Perception and Social Behavior

**DOI:** 10.3389/fpsyg.2019.02599

**Published:** 2019-11-21

**Authors:** Patrick Connolly

**Affiliations:** Counselling and Psychology Department, Hong Kong Shue Yan University, North Point, Hong Kong

**Keywords:** object relations, free energy principle, integrative clinical systems psychology, systems theory, social perception, psychoanalysis, development

## Abstract

Friston’s (2010) free energy principle (FEP) offers an opportunity to rethink what is meant by the psychoanalytic concept of an object or discrete mental representation (Ogden, 1992). The significance of such objects in psychoanalysis is that they may be superimposed on current experience so that perceptions are partly composed of projected fantasy and partly of more realistic perception. From a free energy perspective, the psychoanalytic (person) object may be understood as a bounded set of prior beliefs about a “platonic” sort of person that provides a free energy minimizing, evidence maximizing, hypothesis to explain inference about – or dyadic interactions with – another. The degree to which realistic perception supervenes – relative to a platonic person object – will depend upon the precision assigned to the sensory evidence (concerning the person) relative to the prior beliefs about a platonic form. This provides a basis for not only explaining projection and transference phenomena but also conceptualizing a central assumption within the object relations psychoanalysis. As an example, the paper examines the Kleinian theory of split good or bad part objects as affectively organized generative models (or platonic part-object models) formed in early infancy. This also provides a basis for building on work by Kernberg (1984, 1996) by conceptualizing the role of the part object(s) in a continuum of reality testing, from mild errors in perception that are relatively easily corrected, through borderline affective instability and frequent shifts between part-object experience, to psychotic failures of reality testing, where Friston et al. (2016) proposed that aberrant precisions bias perception to high precision false beliefs (here cast as platonic part objects), such as stable perceptions of others (and possibly oneself) as persecutory agents of some sort. The paper demonstrates the value that the history of clinical insights into psychoanalysis (including object relations) and a system-based approach to the brain (including the free energy principle) can have for one another. This is offered as a demonstration of the potential value of an “Integrative Clinical Systems Psychology” proposed by Tretter and Lo¨ffler-Stastka (2018), which has the potential to integrate the major theoretical frameworks in the field today.

## Introduction

A broad field of study in psychoanalysis focuses on objects, referring to a “mental” object and was described by Ogden (1992) as a discrete mental representation. In theory, objects could refer to mental representations of anything in our lived experience, but the field is primarily concerned with our mental representations of people, whether there are people around us, people who have been important to us in our past, or even ideal or prototypical people who exist only in our imagination.

There are a couple of key reasons that our mental representations of people are worth studying. One reason is that our social behavior may be very influenced by those representations. The way we see people (either people in general, or specific types of people like authority figures, or specific individuals) may strongly influence our behavior toward them. Of course, many factors play a role in any given (social) behavior, including our aims toward others, strategic goals we are trying to achieve, our emotions, and a host of contextual factors. But, a key assumption of object relations psychology suggests that those representations do play quite a foundational role in our behavior, our aims toward people to some extent, and the emotions we feel about them.

Another key reason for interest in mental representations of people is because such representations may be quite different from the reality of the people that they are supposed to represent. Object relations psychologists suggest that a lot of our apparently irrational, self-destructive, or maladaptive behaviors – particularly in social contexts – may make perfect sense when we can see that people are acting consistently with their (often inaccurate) inner representations of people.

Following Kernberg (1965, 1987), a person’s reaction to another person’s behavior is potentially the result of two different components (Kernberg’s idea was primarily applied to counter transference, or how therapists perceive clients, but logically applies to any interaction). The first of these is a “realistic” response to the person’s behavior toward us, including their attitude and emotions toward us. In other words, someone’s behavior might make me feel angry because it typically produces anger in others too, for example, where the other person insults me. We might think of my angry response toward them as a compatible or “rational” response to the other person’s behavior in such a case.

However, my response may also be driven primarily by “fantasy” or the influence of my own mental representations rather than by a realistic perception of the stimulus^1^. In other words, I may have a response to the other person’s behavior that few other people would have, or my response would be much more (or less) intense than other people’s, beyond a level of what could be described as culturally or statistically normal. An example here would be an angry response to another person whose behavior would not ordinarily cause others to behave angrily: another person makes an inoffensive joke that does not really make any reference to me (or my social identity) in any way.

Object relations psychoanalysts (such as Klein, 1946; Kernberg, 1965; Ogden, 1992) tend to suggest that this kind of error in perception is ubiquitous. In other words, at all times, our perception of people may reflect a combination of both the realistic perceiving and some amount of “representation-driven” perceiving. All that vary are the relative *extent* (or ratio) of the realistic versus representation-driven perceiving. For most people, the relative influence of realistic versus representational perceiving varies from one situation to another. However, there are a number of conditions in which we may think that some people regularly have a much stronger influence of representation-driven perception. One instance may be personality disorders, such as borderline personality disorder or paranoid personality disorder, where perceptions of other people are mostly negative. An even more extreme example may be schizophrenia, where a person may remain entirely convinced of seriously hostile intentions of almost everyone around them, despite being exposed to a large amount of information that might appear to contradict such a perception. In each of these cases, the perception driven by the internal mental representation appears less responsive to the information available.

However, regardless of whether we are focusing on typical or atypical social perception, an important question raised by the above account is how such a “quantitative” description (in other words “more” or “less” based on available information) is formalized, and in a neuronally plausible way, which also fits the phenomenology we observe. This is computational in the sense that it describes an outcome (the perception of a person) as the result of two processes (realistic versus representation-driven perception) that appear to operate in opposition to one another, such that the result reflects some relative ratio of both. A computational account would require that the processes be specifically defined as quantifiable terms in an equation that precisely specifies the relation between them.

Friston’s (2010) free energy principle (FEP) as a regulatory principle of biological organisms, including brain processes, offers precisely such a computational expression of the relative influence of informational inputs to the brain and how they are acted upon by the existing mental representations of persons encoded in its neural networks. This paper will outline a free energy principle account of person perception, showing how it is computationally efficient for the brain to encode a prototypical model of what a person is. This prototypical model then exerts a theoretically quantifiable influence on conscious perception. The focus then shifts toward showing how object relations theory in psychoanalysis can make use of this FEP account to demonstrate the unique contribution it can bring to formalizing social perception. The paper uses the example of good and bad part objects found by Klein’s (1946) foundational work in object relations and suggests that these could be understood as distinct affectively organized generative models that play a role in social perception and that come to the fore in emotionally intense experiences. Next, this account of affectively organized part-object models is applied to both borderline personality disorder and schizophrenia to show how this reformulated object relations approach might provide additional explanatory power to current computational FEP-based approaches to these psychiatric conditions. Finally, it is suggested that the formulation in the paper attempts to demonstrate the potential value of Tretter and Löffler-Stastka’s (2018) call for an “Integrative Clinical Systems Psychology” that has the potential to integrate existing major theories of psychology with system-based approaches. We begin with laying out a formal free-energy principle account of person perception.

## A Platonic Person Model Approach to Free-Energy Principle-Based Social Perception

A FEP account of information processing proposes that the physical structure of the brain constitutes a generative model of its environment that actively infers the causes of its sensory inputs and specifies a prior prediction of inputs. The organism (and the brain) acts according to a regulatory principle, which minimizes the differences (more correctly the Kullback-Leibler divergence, or “free energy”) between the prior prediction of the generative model and the posterior likelihood of the inputs^2^. This minimization is achieved either through the Bayesian updating of the generative model or by an action, which alters the inputs in line with the predictions of the generative model. In this way, the free energy in the perceiving system drives both behavior and learning (i.e., belief updating).

A more complete account of free energy minimization rests on mathematically decomposing free energy in a number of ways. First, free energy can be decomposed into expected energy minus entropy. This means that minimizing free energy conforms to (Jaynes) principle of maximum entropy (Banavar et al., 2010). A more intuitive decomposition splits free energy into complexity minus accuracy. This means that minimizing free energy is equivalent to providing an accurate explanation for the sensorium in a minimally complex way – in accordance with Occam’s principle (Maisto et al., 2015). Finally, free energy can be expressed as an evidence lower bound minus the log evidence for the generative model. This decomposition means that minimizing free energy reduces the bound (to ensure that model evidence is maximized). This is sometimes referred to as self-evidencing (Hohwy, 2016). These decompositions are mathematically equivalent; however, the decomposition into accuracy and complexity will figure prominently in the present discussion and is explained next.

The learning (belief updating) process described above always moves in the direction of greater accuracy of the model while minimizing complexity; namely, an oversensitivity to typical changes in inputs. In other words, my generative model of the world grows in accuracy with experience, but once it becomes too accurate (in a sense over-fitted to the data), it is becomes sub-optimal in that relatively small shifts in the state of the environment can now generate larger amounts of free energy (or prediction error). Therefore, it is computationally efficient for our generative models to be abstracted from our sensory experienced to some extent, so that they maximize accuracy while minimizing oversensitivity in typical changes in inputs (Friston, 2010; Hobson et al., 2014). Friston (2017, personal communication) suggests that this would be enough to explain the emergence of a mental representation (generative model) of a “platonic” person^3^ encoded within the structure of the body and nervous system:

“… a prior belief about a … ‘platonic’ sort of person provides a free energy minimising, evidence maximising, hypothesis to explain inference about – or dyadic interactions with – another. In other words, having a particular hypothesis or platonic person in mind allows you to immediately explain prosocial cues in an accurate and parsimonious fashion. The parsimony afforded by the object minimises complexity and thereby free energy.”

This computationally efficient set of expectations cohering around an abstracted “platonic” model of a person informs our expectations regarding what people do and how they think and behave. In terms of how this generative model of a platonic person is encoded in the brain, this must of necessity refer to distributed networks of neural relationships within a multi-level hierarchy of organization, consisting of faster sensory-level priors and increasingly slower, more abstract integrative priors extending through the highest levels of cortical organization^4^.

## A Free Energy Formulation of Reality Versus Representation-Driven Perception

The FE formulation of the platonic object as described above now allows for a formal statement of the relationship of reality versus representation-driven perception. Friston (2017, personal communication) describes how this distinction might be formulated in a free energy perspective:

“The degree to which realistic perception, relative to a platonic person object supervenes will depend upon the precision (usually cast as attention) assigned to the sensory evidence (concerning the person) and the prior beliefs about a platonic form. In other words, your posterior beliefs – following an encounter with another – will be a mixture of the object prior and the likelihood that the object is behaving in a way consistent with that hypothesis – or an alternative object.”

In other words, this quantitative relation between reality and representation-driven perception is described in terms of probability. The posterior belief, which reflects our experience of a person, is partly determined by the information we receive, and the relative extent to which it matches (or does not) our existing prior. However, it is not just the information itself that determines this relative probability; as indicated above, it is also the precision afforded to the prior prediction. What precision means here, is the confidence assigned to the higher-level predictions of our generative model – if its high relative to the precision afforded to the sensory evidence, discrepancies with the sensory evidence will be attenuated to some degree, and vice versa.

This description can be thought of intuitively in terms of a formal similarity with gravity. In other words, our prior predictions exert a kind of influence on the perceived information, where it is stronger we tend to perceive our “representation” (object prior) and where it is weaker, we may perceive more of the reality, in so far as it is different from our inner representation. Friston (2017, personal communication) suggests:

“The ‘gravitational pull’ of the object is, exactly the relative precision afforded to the prior hypothesis of the object, relative to the sensory evidence. In fact, mathematically, the equations that govern the posterior expectation have exactly the form used in Newtonian mechanics and gravitation.”

The implication of this perspective is that our perception of people will always tend to some extent toward our platonic model of the person.

If we assume a hierarchical recursive development of the platonic model of the person (Connolly and van Deventer, 2017), we understand each new level of organization of this model to be constrained to some extent by what came before. In other words, our earliest experiences of people in a sense lay the foundation for the future development of the model.

The account of errors in perception offered thus far is not formally similar only to psychoanalytic theory. It is an established idea in cognitive theory that our social perception is shaped by schematic representations of people, which may lead to inaccurate information processing and maladaptive behavior. It may well be that an integrative clinical systems theory (Tretter and Löffler-Stastka, 2018), which is potentially able to incorporate a system paradigm such as Dr. Friston’s work, may well be able to integrate these different theoretical perspectives, and more is said on this toward the end of the paper. However, the value of the field of psychoanalysis to the future growth of an integrative clinical systems paradigm lies in its sizeable literature of clinical insights that offers the possibility of better models, or descriptions, in this case of our person perception. In this regard, one of the most immediate contributions that psychoanalytic theory can make to the current FEP formulation of person perception is the observation that most minds contain more than one such platonic person object.

## Multiple Objects

While the body of object relations theory may describe many different taxonomies of person objects, for the sake of clarity, this article will focus in detail on just one in order to unpack how a formal description might work and to examine its potential implications. In Klein’s (1946) seminal text “Notes on some schizoid mechanisms,” she proposed that in the earliest months of an infant’s life, the child did not yet have an integrated (platonic) person object with which to perceive people as unitary, complex objects. Rather, we perceived only “part” objects, which are incomplete and fragmented representations of people, such that we might perceive a particular person at times as one part object, and at other times, a different one. She focused on part objects defined by good and bad experiences. Primarily, she focused on the child’s experience of the mother’s breast, defining the “good breast” as a founded upon a good experience of the breast as satisfying and pleasurable. By contrast, the “bad” breast was founded upon unsatisfying, frustrating, or withholding experience, into which the child projected their hostile emotions, borne of those experiences. She then described how, beginning from roughly 6 months of age, the child began to integrate those part objects into a whole object representation and perceives the mother as a whole person (Klein, 1946).

While Klein focused on the breast as a part object, it is broadly understood that these representations, fragmented as they are, are nonetheless part representations of different aspects of persons, though they are not yet perceived as belonging to the same object, the whole person. In other words, my experience of the bad mother part object is not yet integrated with my experience of the good mother part object. These must reflect different prototypes of platonic persons that are encoded as distinct generative models at this early stage of development.

The idea that an organism can encode distinct generative models for different “others” has been described by Isomura et al. (2018, unpublished). In their paper, they describe a theoretically and neurobiologically plausible model whereby a bird may fit sensory inputs from other birds under distinct generative models. In this way, they may “know who they are communicating with,” which allows for appropriate inferences within complex social environments.

While the formulation of Isomura et al. (2018, unpublished) might support the idea that we have different generative models for different “people,” it should be made clear that Klein’s theory of the good and bad object is not referring to different individuals (though it may initially), so much as it refers to different *types* of person or rather different platonic persons. In this case, these distinct platonic objects are founded upon different emotional experiences (pleasure and satisfaction versus dissatisfaction and frustration).

An indication of how such models may build upon a base of emotions, Panksepp’s (1998) work on affective systems of mammalian brains is useful here. Panksepp described seven affective (command) systems common to mammalian brains, activation of which was associated with observable affective states and related behaviors. He presented these as in terms of core affective descriptions such as RAGE, LUST, or SEEKING and described the neural systems that appeared related to each of these. While Panksepp (1998, 2010) has offered a lot of evidence for his claims, there have nonetheless been criticisms, including some regarding the complex expression of human affect (Barrett et al., 2007). However, Panksepp (1998) did express the hope that 1 day the role of the affective systems would be understood within a broader system-based understanding:

“The basic emotional systems may act as ‘strange attractors’ within widespread neural networks that exert a certain type of ‘neurogravitational force’ on many ongoing activities of the brain” (p. 3).

Here we see that affective systems may have their own “gravitational force” that entrains^5^ the activities of the brain within their ambit, that is, at first (if Klein is correct), a stronger attractor than a platonic person object (which does not yet fully exist in the infant’s brain). Fitting this description within a hierarchically recursive scheme of the nervous system, we could say that the seven affective command systems described by Panksepp have a tremendous influence on higher-order functioning of the brain, acting as constraints on superordinate levels of processing, such that our earliest experiences of people may be updating generative models that were originally distinguished by affect, just as Klein suggests.

## The Emergence of a Dominant Platonic Person

Around 6 months of age, Klein (1946) suggested that the child began to integrate these affectively defined good and bad objects within a “whole-object” representation in which the mother is perceived as a unitary person. She also proposed that there began a meaningful shift in the affective relationship with the whole-mother object that tended to move away from extremes of persecutory anxiety, rage, and intense idealization, toward a more ambivalent relationship characterized by guilt and reparation. This also marks the beginning of our capacity for more realistic object relating. We might say that the gravitational pull of the generative model that is organized by experiences of the whole object slowly begins to exceed that of the part objects that were organized by the affective command systems. However, this is better described in the sense of a recursive hierarchy (Connolly and van Deventer, 2017), where perception is originally entrained by the affective systems but later both perception and the affective systems come to be entrained by the increasingly stable object organization, which has emerged as a superordinate level of organization to that determined by the affective systems, though is still constrained by them.

**Figure 1:**
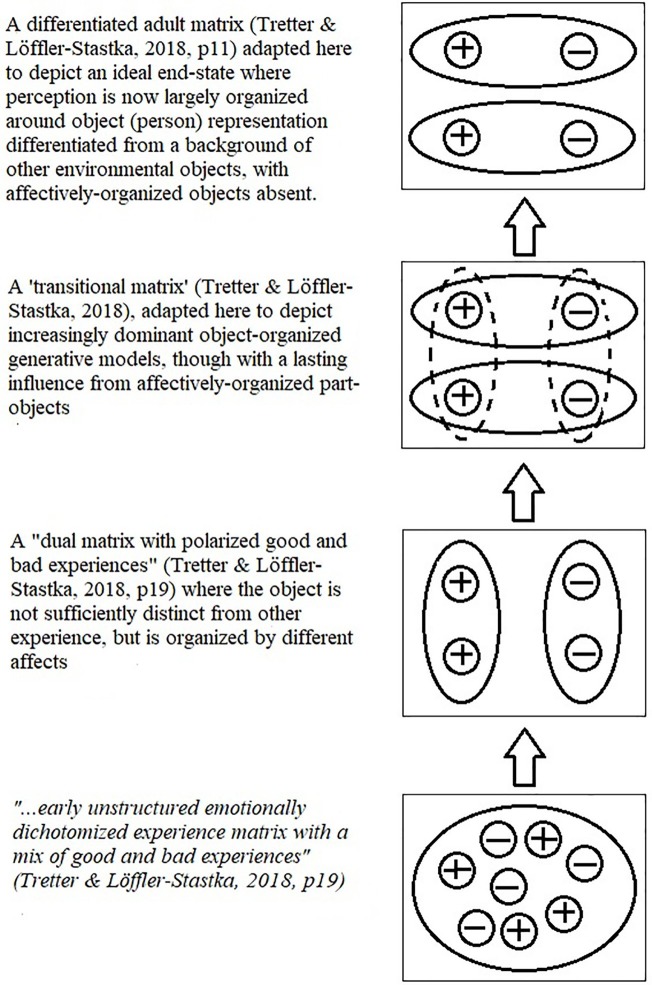
The emergence of person object representation differentiated from other representations, from a lower level of affectively organized part objects (adapted from Tretter and Lo¨ffler-Stastka, 2018, p. 11, with permission from the copyright holder).

This state of affairs is represented in the hierarchical matrix in Figure 1, which is adapted from one found by Tretter and Löffler-Stastka (2018), which displayed the core concept of object relations theory (Kernberg, 1976) as a matrix, though their figure included a description of the emergence of self from the environment, while the present figure focuses on describing the emergence of an object representation from a background of other experiences (including of the self), through a recursive development^6^.

The view being given in this paper is that prior to the emergence of a dominant platonic person object, there are some number of distinct generative models (that predict sensory inputs), which are largely organized by the affective systems (depicted in Figure 1 by the second layer from the bottom). The question here is how a dominant platonic person object comes to emerge.

**Figure 2:**
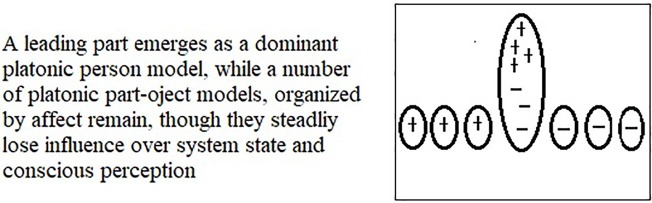
The emergence of a dominant platonic person model (integrating positive and negative affects) as a leading part from a layer of part-object models organized by affect.

Here, we can borrow terminology from systems theory by describing the emergence of distinct generative models for person perception as a progressive segmentation occurring within the broader generative model, where one of the parts comes to lead, through a feedback loop with the sensory data, until it is dominant, a state of affairs depicted in Figure 2.

It is important to note that what is being proposed is that dominant platonic person representation has been built upon “part objects” that were previously affectively organized. It might be tempting to suggest that it is a model built upon one particular affective system that comes to dominate. For example, since we think that maturation involves an increasing ability to perceive people without much apparent emotion, we might suggest that models built upon the SEEKING system steadily come to dominate.

However, it is very unlikely that the dominant platonic person object is founded on the activity one particular affective system. Rather, given the phenomenology we observe in people, it must be a more complex mixture of the pre-existing generative models (the part objects).

From the formal perspective of minimizing free energy – or maximizing model evidence, the hierarchical assembly of parsimonious models of the (prosocial) world through our development can be considered in the light of Bayesian model selection or what is called “structure learning” (Gershman and Niv, 2010; Tervo et al., 2016; Isomura and Friston, 2018). In other words, one can build more comprehensive (deep) generative models that have greater evidence (i.e., accuracy minus complexity) by adding layers or rearranging part objects into more complex (or deeper) wholes. These operations of hierarchical assembly, for example, “split” and “merge” operators, figure prominently in machine learning and statistics – and may provide a nice metaphor for the merging of part objects into more complex (or more dominant) objects or, as will be shown later, the “splitting off” phenomena seen in psychoanalysis.

This tendency toward hierarchical assembly of existing part objects may also imply that the emotions that we tend to experience often during early development tend to have a greater organizational influence (in the form of constraints) on the dominant platonic person model. For example, frequent experiences of PLAY affects in our early development are likely to influence the platonic person object toward perceiving people as fun, while frequent experiences of FEAR are likely to influence it toward perceiving people as dangerous. Each new experience characterized by these emotions increases the influence they have over our social perception. Over time, where the environment permits, these are likely to become stable, self-organizing perceptions of people^7^. This description also suggests how our dominant person model may form templates of different types of persons, partly constructed from different combinations of pre-existing part objects.

## The Persistence of Primal Objects

The section above has offered a theoretical formalization of Klein’s (1946) assertion that we come to perceive people as whole objects, so that (to some extent at least) we perceive that a person is the same person regardless of the emotions we have toward them. However, the capacity to “split” our perceptions of people around us into good and bad objects appears to be an ongoing phenomenon well into adult life, especially in particular situations, in which it is understood as a “splitting” defense. We may perceive a competitor in an intense rivalry as “all bad,” or a new lover as “all good,” or split the representation between people, for example, two teachers at school, one all good, and one all bad. These states supposedly reflect the persistence of the good and bad part objects as “latent” objects in the organization of the psyche that may nonetheless come to the fore in situations that activate them.

We might say that the dominant platonic person object gains profoundly greater influence over perception relative to the generative models of the affective (e.g., good and bad) part objects, and it is plausible that those part objects remain present as an influence in the nervous system provided that the connections that encode them experience reconsolidation at least occasionally.

The influence such “primal” generative models have on conscious perception^8^ may vary from extreme to fairly subtle. At the extreme end are experiences of the most intense emotions, where we seem to experience almost nothing else but that emotion, with little higher thought process. An example is a man going through an acrimonious divorce process who encounters his ex-partner at a shopping center, accompanied by a new lover. He later describes himself as overwhelmed and rooted to the spot, at that moment feeling as if the universe had just shattered in some way, as everything else faded into the background and he only saw her laugh, her hand on her new partner’s arm, and experienced her only as a terrible beautiful thing that was tearing his body apart from the inside. Shortly afterward, a more normal thought process resumes though he feels shaken and very distressed.

We can suggest that a part object has pulled perception entirely within its event horizon, and the dominant platonic generative model seems to have lost all influences on perception during this experience. At that moment, the man does not perceive his ex-partner as a “person” at all, but only as some surface sensory characteristics, distressing interoceptive sensations and an inarticulate sense of persecution.

The key point being made here is that there may already be a pre-existing generative (part-object) model, which represents the best prediction of the combination of sensory and interoceptive input at this present moment. The most typical hypothesis among object relations thinkers is usually that there are experiences of early childhood that were of similar emotional valence, which are activated by the contemporary experience. For example, feelings of abandonment (likely related to activation of PANIC/GRIEF affective functioning, as described by Panksepp, 2010) connected to several early experiences of the man’s mother regularly going to work in the morning after only a few months of maternity leave, chatting with his father as she walked out the door, and other similar early experiences that organized around those emotions.

The reason for why ordinary person perception seems to be suspended may be partly due to the fact that the thought processes are state dependent to some extent, and most people have very limited experiences (and generative models) at such intense emotional valences^9^. These state-dependent thought processes, emotions, and body feelings may also compete with more ordinary person perception for activation of shared networks, such as described by Oosterwijk et al. (2012).

A further consideration relates to Freud’s concept of conflict, where aspects of our psyche no longer undergo normal development due to their generating too much conflict during development and remaining repressed (Freud, 1912/1963, 1915/1963). This has been formally described by Hopkins (2016) and elaborated by Connolly (2018), as a situation where alternative plans of action generate similar high levels of expected free energy. Through development, the “loser” of this contest becomes progressively less able to determine high-level conscious experience. In this way, the child’s distressing experiences related to feelings of abandonment (PANIC/GRIEF affects) when their mother left for work may likely lead to policies of action (such as rejecting the abandoner) that also generate high expected levels of free energy. To resolve the conflict, the superordinate levels of the person’s generative model alter the precisions afforded prior beliefs about policies of action, where these prior beliefs are based on the expected free energy following a particular action. Should the distressing feelings related to PANIC/GRIEF lose the competition, they become less and less likely to be activated in the normal course of affairs, and the dominant platonic person model that emerges through further development is likely to encode this constraint. In this way, the part model becomes a “split-off” remnant that is not integrated into the dominant platonic person model and does not undergo the significant further updating that the dominant model does, though it may come into association with experiences of similar emotional valence (Freud, 1915/1963), which seems to be what happens to the man in the example^10^.

The rubber hand illusion provides a nice metaphor for this sort of process from a free energy perspective^11^. In the rubber hand illusion, concomitant visual and tactile information is supplied *via* stroking a rubber hand, inducing the illusion that the hand is part of one’s body. The most common explanation – for this illusory body ownership – is that the proprioceptive (position) sensory information that is attenuated (i.e., ignored) by reducing its precision (Paton et al., 2012; Seth, 2013; Zeller et al., 2015). This enables a low free energy explanation for the coherent visual and tactile information under the belief that “I only have one right hand.” In short, the high level prior beliefs about the part objects that comprise my body can have a profound effect on the way in which evidence is accumulated for those beliefs, under active inference. In this way, the attenuated proprioceptive sensory information is akin to the split off remnant described in the paragraph above, in which it is no longer consciously experienced in the ordinary state of affairs. Rather, similar to the high-level beliefs about one’s hand, beliefs about a dominant platonic person model come to have greater precision and begin to shift the accumulation of evidence in line with this prior.

This description of the early formation (and splitting off) of the generative part-object model might also offer a hypothesis to explain the dissociated or “de-realized” characteristics of the experience, where the surrounding reality, sense of self, and ordinary thoughts are somehow not perceived consciously. These earliest part-object models formed in early stages of development where functional connectivity is far less developed. For example, in research that later led to a Nobel prize, Hafting et al. (2005) reported the activity of grid cells that provided a sense of place throughout all experience. More recently, Tsao et al. (2018) have shown that cells in the lateral entorhinal cortex encode a perception of time in experience. While we are born with these structures, their successful integration with conscious perception is surely a developmental achievement. It seems possible that the seemingly “derealized” nature of these experiences may result because these part-object models formed before such complex integration has fully taken place. Of course, it may simply be explained rather by the intense emotional valence and demand for network resources meaning that the activity of these orienting systems is temporarily not integrated with conscious perception. However, the present formulation offers an alternative hypothesis, and, of course, both may simultaneously be true.

The above descriptions have referred to situations where primal part models influence conscious perception in an extreme sort of way. However, their influence may run on a continuum down to more subtle influences. This refers to situations where our dominant platonic person generative model is largely engaged in active inference of a social situation, but platonic part models still “drag” the perception in their direction to some extent.

As an example, we could refer to the same man as in the example above, though at an earlier point in his marriage, before the divorce. During dinner, his wife answers a phone call, says it is a work colleague, and steps outside and has a long laughing chat on the phone, leaving the husband to eat alone with their children. The man feels irritated by this, but thinks no more of it. However, he finds he is irritable with a number of his wife’s behaviors for the rest of the evening, perceiving a lack of care or consideration in several behaviors. Only after some reflection does he realize that it began with the phone call.

In this instance, it may be that the platonic part model is activated, but unable to have the same dramatic influence on conscious perception. Instead, its influence on conscious perception can be thought of in terms of the binocular rivalry paradigm as presented by Hopkins (2012) where competing predictions about different stimuli presented to each eye (e.g., whether it is a house or a face) seem to dominate in cycles. Following that example, one can think of the dominant platonic person model as being in competition with the platonic part model to explain the current stimuli (in this case, the wife stepping out of dinner to chat with a colleague). The part model may not be dominant enough to come to define conscious perception as it did in the more extreme example above (or in the binocular rivalry example) but may still generate some lower level free energy within the psyche, which may not be adequately explained by the model dominating perception. This activation of the part model and its accompanying affect sets up a feedback loop where it becomes sustained over the rest of the evening, where the man’s continuing experiences of his wife throughout the evening trigger inferences to explain the negative affect (inferences related to perceiving her behavior as “abandoning” him or not considering him), which reactivates the part model, and so on.

This feedback loop seems to explain how, once we dislike a person, we may often struggle to shift into liking them, particularly when we do not really know why we dislike them. The negative affect emerging from whatever negative part models activated by our experience of that person seems to result in an ongoing process of negative inferences about the person’s behavior that feed back into the reactivation of the underlying part models (if they are involved in the dislike), even though we may never have a conscious perception of what we really feel about the person, and why. Having said this, we do nonetheless have experiences of being able to escape a more transient affectively influenced perception of a person, which is addressed next.

## Dominant Versus Part Models, Not Cognition Versus Emotion

We do have experiences where we have a strong emotional reaction to a person (and an attendant set of inferences), and then seem to have an insight or a new thought about it that seems to reinstate an apparently more objective perception. An example might be encountering a cashier in a supermarket who seems to be surly and unpleasant in handling our transaction and rolls his eyeballs when we drop an item by mistake. We may have a strong negative reaction and make an irrational inference that he dislikes us personally and is attacking us. Then, we suddenly think that it is likely that he has a bad mood (and perhaps often does) or treats almost everyone like that. We may even wonder about what troubles he may have in his life that makes him unhappy. This can seem to make our intense emotional response (and personalized inferences) evaporate fairly immediately.

It is this form of “reality-testing” process that is one of many observations that seem to support the idea that cognition is in competition with emotion to determine our perception, and the potential that higher cognitive process has to influence emotion-driven thought processes. However, it may be that “emotion versus cognition” is not the best distinction to draw in this situation. Rather, we can describe a competition between a highly developed dominant platonic person model and a more archaic, underdeveloped part-object model, to regulate free energy.

The more extreme activation of the part model in the earlier example of the cashier is distinct precisely because the more complex dominant model *fails* to entrain the activation of the part model (for a brief period at least) because this part model has never been integrated within the dominant model for the reasons described earlier in the paper. However, the much greater pull of the dominant model soon reasserts itself. It seems clear that through development, as our platonic person model (and the functional connectivity that underwrites it) ascends in complexity and ever more accurately “recognizes” these states, it entrains our affectively organized experiences more effectively as well and reduces their free energy. Besides this influence of general development of the dominant platonic model, the tendency toward reducing psychological conflict through altering the precisions regarding prior beliefs of expected free energy ascribed to policies of action (Hopkins, 2016; Connolly, 2018) related to part-object models means that they are increasingly avoided as the superordinate levels of the brain hierarchy that encode those precisions also develop. This may underlie a tendency toward reduction of the frequency and duration of intense part-object experiences in life, though it is a journey that may never entirely be complete.

However, in contrast to this tendency, a trend in research into psychopathology has focused on more severe deficits in functional connectivity underlying problems of active inference (rather than the more typical phenomena described above). Disorders such as autism, schizophrenia, and personality disorders have been cast as problems of social inference. The next sections of this paper seek to apply the formulation that has been developed this far to briefly outline the potential it can have to contribute to our understanding to this approach to both borderline personality disorder (BPD) and schizophrenia. In doing so, the author attempts to place these phenomena on a continuum in terms of the relative stability of the dominant platonic person model in perception (or oppositely, the relative influence of part-object perception) and by implication, the level of effective functional connectivity that supports the dominant model. The subtle problems of reality testing described above refer to relatively higher dominant model, low part-object model perception (and relatively more normal connectivity), while BPD (examined next) is cast as more severe problems in maintaining the dominant model that entrains our perception, and schizophrenia representing the most severe problems in entraining part models in perception (and the most severe problems of connectivity)^12^^,^^13^. The distinction between BPD and schizophrenia is given here in terms of functional differences in terms of the level and stability of part-object object perception, and by implication, the level of functional connectivity, though these disorders may have discrete patterns of neurophysiological presentation and aberrant connectivity as well.

This presentation of disturbances in object perception on a continuum of levels of dominant versus part-object perception is both consilient with and inspired by a formulation by Kernberg (1984, 1996, 2004). In his work, Kernberg describes three levels of personality organization on a continuum of reality testing. The most intact reality testing is reflected in merely “neurotic” personality organization, while personality disorders such as BPD and schizophrenia represent more serious and most serious problems of integration and reality testing, respectively. The purpose of the following two sections is to highlight how this continuum of reality testing could be expressed in terms of a free energy formulation focused on the relative influence of dominant versus part-object models.

## Borderline Personality Disorder

A hallmark of the experience of people diagnosed with borderline personality disorder (BPD) is the instability in their perception of self (identity) and others. In psychotherapy, clients diagnosed as BPD may move easily between extremes of idealization and aggression or persecution (these negative responses are often frequent) in their transference responses to psychotherapists, as well as to perceptions of other people in general (Yeomans et al., 2015).

**Figure 3:**
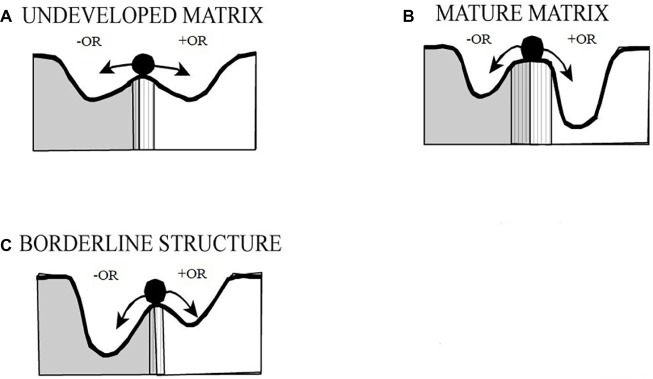
Borderline dynamics of affectively organized object representation (OR), modified from Tretter and Lo¨ffler-Stastka, 2018, p. 11, with permission from the original author and copyright holder, Thieme Publishing. These figures show basins of attraction for positive and negative affect for typical early development **(A)**, typical mature development **(B)**, and dynamics of Borderline structure **(C)**.

Figure 3 is again adapted from a figure found by Tretter and Löffler-Stastka (2018). It presents a development of affectively organized object representation in both typical developed configurations and BPD configuration. In the first infant stage, the system’s current state (represented by the ball) can more easily move between extreme positive and negative basins of attraction, formalizing a state of instability within this “semi-quantitative” model. The deepening of these basins during development reflects the tendency toward greater affective *stability* (not intensity), with a reduced tendency to move toward opposite poles. The growth of the central barrier through development (marked with vertical lines) could be described as formalizing the increasing influence of dominant generative models of self (Tretter and Löffler-Stastka focus on self-representation with regard to BPD) and of others – the dominant platonic person models described here. As these dominant models begin to grow in influence over the system state, there is a reduction in the tendency to move toward extremes of positive and negative affectively organized states, or between them. Tretter and Löffler-Stastka described BPD as an intermediate position where the boundary between states is less developed (and negative states are a stronger attractor than positive ones).

Their model focuses on “object-related” self-presentation, and while the present paper has not addressed the dynamics of self-representation, the theoretical account that has been put forward in this paper is compatible with the formal account found in their article. Whether it may be due to a predisposition toward subtle problems of functional connectivity that limit the development of highly complex person models^14^, or due to an excess of negative affective experience during early development that similarly places constraints on the functional connectivity underwriting the potential complexity of our platonic person model^15^ (or both), we may suggest that the dominant platonic person model is less well developed, and less complex, and ultimately less able to entrain (predominantly negative) part-object models in conscious perception of other people.

## Schizophrenia

Impairment in reality testing of perception or beliefs is the key defining characteristic of psychotic disorders such as schizophrenia. This often manifests in delusional thought content, which appears relatively impervious to any contradictory information, particularly persecutory delusion (American Psychiatric Association, 2013). While people diagnosed with schizophrenia may have a variety of delusional thoughts over their lifespan, there is often a core (typically persecutory) delusion that never shifts, even among those who successfully maintain a residual phase following some number of breakdowns, though it may reduce in importance to the thought process of those who are relatively well. These core persecutory delusions may often be bizarre, such as perceptions that other people are demons or witches, or similar. These symptoms may occur against a backdrop of a relative poverty of thought, particularly in chronic cases with a history of frequent hospitalizations.

Computational approaches to neuropsychiatry such as those using a free energy principle framework have approached schizophrenic symptoms as rooted in a deficit of functional connectivity and hence of complex generative models (Montague et al., 2012) and have approached persecutory delusion (PD) as aberrant social perception related to impairment of generative models of others (Diaconescu et al., 2019). These accounts may offer a satisfying account of the failure of more realistic perception, and Friston et al. (2016) suggested that the relative persistence of false beliefs (delusion) in schizophrenia reflects an increased precision given to prior (false) beliefs in response to failures of attenuation of sensory information. However, what their account does not clarify is what sorts of inferences are like to come to the fore in PDs, or rather what the reason is for the specific affective or thematic nature of those false beliefs. Certainly, the schizophrenic person’s inferences that external influences are controlling their experience and behavior as described in their paper are likely to give rise to negative affect and inferences of persecution, which is of course possible. However, the present paper offers an alternative suggestion in which the specific affective and thematic characteristics of the delusional experience are the consequence of *pre-existing* models, which offer the best active inference for the abnormal conditions present in the brain.

An established idea in psychoanalysis is that psychotic experiences of PD may in some sense be founded on split-off persecutory bad objects in the Kleinian sense (Klein, 1946; Segal, 1964). Within the current formulation, we might suggest that the failure to maintain a highly developed platonic person model that regulates free energy in daily encounters, the system falls back on less well developed, but meaningfully established part-object models (such as a persecutory bad object) that require far less effective connectivity to operate.

Though these part models are far less able to reduce the free energy of the system (they integrate far less sensory input than typical dominant models do), in a sense they become “the only game in town” as the only regulatory generative model available that can explain away the person’s social experience. This may go some way to explaining the relative intransigence of such core PDs (formally described as increased precisions of these deep priors), as they become the central foundation of the person’s social perception^16^ and even start to undergo some development and updating themselves (e.g. the patient forms detailed verbal structures around them), though this is clearly limited by the general constraints offered by the problems in connectivity.

This also goes some way to explaining their persecutory character. We might explain this in a narrative way. First, during the long, distressing prodromal period, the inability of the dominant platonic model (and perhaps self- and various other models as well) to integrate experience^17^ leads to increasing free energy. This progressive failure of the dominant model and escalation of free energy and negative affect likely activates negative affective part objects (which form in similar circumstances early in our development) in a feedback loop, and they begin to gain dominance in our conscious perception as they increasingly become the best (or only) available inference about our (social) experience. As the person reaches the more acute phase, the increasing failure of dominant models and ascendance of part-object perception drives the magnitude of the derealization described earlier in the paper (in the “extreme” example of the man who sees his ex-partner with a new lover), an overall situation which is all the more fundamentally traumatic as it does not go away after a short time.

The present formulation of psychotic phenomena supports the psychoanalytic perspective of the compensatory or defensive nature of the positive symptoms of psychosis, first articulated by Freud in “The Neuro-Psychoses of Defence” (Freud, 1894/1962) and now supported by the description by Friston et al. (2016).

The remainder of this paper is given over to a discussion of the implications and contributions of the current paper, as well as the problem of evidence of the current formulation as well as possibilities for future research.

## Contribution and Implications

In order to make sense of any implications of the current paper, it is necessary to clarify what the specific unique contribution it aims to make to existing literature. The present paper is offered as theory. In doing so, it builds on existing theory. The theory put forward in this paper is built on ideas within object relations psychoanalysis, specifically on Klein’s (1946) theory of splitting, good and bad part objects and whole object integration. It is also built on Kernberg’s (1965, 1987) description of realistic and fantasy components of object representations and makes use of his (Kernberg, 1984, 1996, 2004) work on a continuum of levels of reality testing through neurotic, personality disordered and schizophrenic personality organization. What is uniquely offered here is an attempt to state these theories in such a way that they fit within a newer theoretical paradigm, which could broadly be subsumed under the free energy principle. In this way, it is built on Friston’s (2010) theory of the free energy principle as well. So, the uniqueness lies in the attempt to marry these theories.

The value of this union from the perspective of psychoanalysis could be said to be twofold. First, the value of stating the psychoanalytic theory in terms of Friston’s free energy principle (FEP) theory lies in the value of the FEP theory itself and the contribution it brings to psychoanalytic thinking. Second, the value of stating the psychoanalytic theory in this way also lies in connecting it to a broader system-theory perspective of the world, within which the FEP theory could be said to reside as well.

First, the value that comes from the FEP is that it articulates a neuronally plausible process theory regarding regulation and message passing within the nervous system (Friston et al., 2017) that offers anatomical constraints on those processes, which can and have been assessed empirically (Parr and Friston, 2018). This allows a stronger explanation of the phenomena described by psychoanalytic theories than the psychoanalytic theories themselves, which have historically not been adequately connected to neurophysiological processes, nor even to other psychoanalytic ideas. This lack of a functional psychoanalytic metapsychology can be demonstrated by asking the question: why do objects form in the psyche? If we follow Freud’s partially failed energic explanation (Connolly, 2016, unpublished) we could say objects form because they bind free-floating energy, but we would be unable to adequately link Freud’s energy with neurophysiological process. If we take Klein’s (1946) suggestion that the formation of objects manages anxiety, then we link it to an abstract emotional construct, but not to neurophysiological process. But if we follow a free energy explanation, we say that objects form because they minimize free energy, through maximizing accuracy of generative models as parsimoniously as possible. We are then on firmer ground, as this explanation is rooted on the neuronal foundation described by Friston’s (2010) work.

The field that is expanding around the free energy principle is itself embedded within a broader framework that is well described as systems theory, which is the second benefit of the union of theories offered in this paper. A fuller description of systems theory and its potential value to psychoanalysis can be found by Connolly and van Deventer (2017) but can be heavily summarized here as saying that a system view of the world is a hierarchical one, where system is superimposed on system and so on.

This hierarchical perspective can be seen in the view expressed by Tretter and Löffler-Stastka (2018) when they call for an integrative clinical systems psychology:

“… The crucial term ‘system’ is defined as a set of elements and a set of relations (structure and connectivity), … In line with this definition, a system can be characterized simply by the term ‘structure’ or by the popular expression ‘network’ (nodes and edges) as it is a network with boundaries. Or, with other words: a living system is a network (or structure) with boundaries. Properties of systems are states (e.g., equilibrium, non-equilibrium) and processes, some of them have goal-directed functions as a subset of activities. … Systemic exploratory methodology basically implies to zoom into the micro-level of the subject of study, not forgetting the context and also to zoom out to the macro-level without forgetting the details. If we zoom out of the detailed consideration of elementary functions of the mind to a more holistic view we will refer to several holistic models that also will provide a diversified understanding of mental processes in context of clinical issues” (p. 7).

Their work suggests that we might define a system as a set of elements and relations between them, where if we “zoom in” to higher resolution we see that each of those elements is itself composed of a system of elements and relations, and so on. The key point they make is that most major theories of psychology might be represented in an abstract description of this form, where theories are not “floating” in an abstract space where they merely have a heuristic or *ad hoc* role in explaining research findings but rather are embedded in a larger superstructure. In this way, different theories (including at different levels of organization in the person) can be integrated with one another. This offers the hope of convergence in our theoretical work, rather than the seemingly endless divergence of theory that has taken place in the field of psychology.

The free energy principle and the body of theory that are growing under its ambit fit the bill of a system-based theory that offers clear system principles and a basis for hierarchical organization of systems and sub-systems. The FEP paradigm can “zoom down” to show how the FEP-based organization of living systems is founded upon inorganic processes (Friston, 2019, submitted) and equally, zoom up to social, cultural, and environmental systems that entrain living systems (Connolly and van Deventer, 2017; Badcock et al., 2019).

Specifically, in this paper, this hierarchical embeddedness of the processes described lies in the foundation of the affective systems described by Panksepp (1998) and how their cortical influence in the form of part objects steadily becomes entrained by a history of social interaction, which comes to form the dominant platonic object.

This integration of the theory of objects and part objects with a system-based FEP perspective now also allows integration with the psychoanalytic principle of conflict, which was integrated with a FEP perspective in the work by Hopkins (2016) and Connolly (2018). This has allowed the current paper to offer a conceptual account of how conflict can lead to the splitting off of part objects and thereby integrating these different psychoanalytic theories rather than leaving them separated across the gulf of their respective Freudian and Kleinian paradigms. Through a steady work of application of system-based ideas in this way, a new psychoanalytic model of the mind may eventually emerge.

Beyond these very broad implications, the integration with a free energy principle account has more specific implications for how we conceive of objects. Some of these are highlighted next:

1. A part object is here described as a generative model. This means that it reflects a distinct anatomical expression with a Markov blanket. This itself has a number of implications. One key one here is that it “tries” to maintain its own existence and avoid destruction (phase change). In other words, one could state it intuitively as saying that the object has a “life of its own.” This also means seeking to accumulate evidence for its own existence. This supports Freud’s (1912/1963) idea that we appear to seek transferences out (try to apply them to each new person we meet).
2. Part objects must have some success in predicting situations, or people’s behavior, or they could never be sustained. This might explain the common preference for entertainment that portrays people in “archetypal” ways. In this way, part objects can accumulate evidence. This would also be true for a common preference to “want” to see others in distorted ways, for example, seeming to “relish” describing someone as a villainous person.
3. While part objects may be “starved” somewhat, in the sense of being prevented from accumulating evidence in some way, they are difficult to get rid of, for the reasons indicated in the previous points. However, they may be entrained, which essentially means being increasingly merged with a more dominant, integrated model. Practically, this could mean the further development of the dominant model (such as through mentalization), as well as recognition, insight, and perhaps also acceptance of these relevant qualities in oneself and others.
4. Recently, Ramstead et al. (2019, submitted) argued that hierarchical generative models do not so much have the characteristics of representation as they do of control. That means that part objects, as well as dominant objects, are not just representations but rather realize the function of control in the psyche and integrate relevant actions in a sense as well. As Ramstead et al. (2019, submitted) suggested: “… ‘perceptual inference’ is just one moment of the policy selection process in active inference under the FEP, namely, state estimation. The issue we want to press here is that the active inference framework implies that perception is a form of action, that is, action and perception cannot be pulled apart …” (p. 2). This means that part objects are perhaps best not thought of just as representations of perceptual memories, unless we think of memories as control mechanisms in the same way as well.

These potential implications are just a beginning, and further implications may be uncovered with further progress.

While the integration of these psychoanalytic theories with the free energy paradigm has many tangible benefits for the body of psychoanalytic theory, the question might well be asked what they offer to the growing field within the active inference and the free energy. As stated earlier in this paper, the central value of the psychoanalytic literature is a long history of observations and clinical insights that can help direct research. In this case, it may generate interest in research into the role of part objects of the kind described here, in perception.

## Evidence and Future Research

A critical problem with the current paper is the lack of empirical evidence for its central claims. The central claims are as follows:

1. Part-object models organized by affect typically exist in human nervous systems since early childhood.
2. They may sometimes not be entrained by a dominant model (perhaps due to conflict), and a competitive relationship may exist between such split off part-object models and dominant ones in order to determine the process of active inference.
3. Increasing levels of influence of part objects (and corresponding decreases of influence of dominant models) on a continuum from transient emotions, to personality disorder (e.g., BPD) and to schizophrenia, in order, probably due to problems in connectivity which underlie dominant models.

As such, none of the research referred to in this paper directly proves these core hypotheses.

Rather, the present paper has taken the form of an argument and has used research findings along the way to support specific points and assumptions being made during its course. For example, the claim that affect may play a foundational role to object formation is supported by making reference to Panksepp’s (1998) work on affective command systems. Claims regarding the role of connectivity in reality testing were supported with empirical findings regarding connectivity in transient emotional experiences (Eryilmaz et al., 2011), borderline personality disorder in terms of genetic predisposition (Witt et al., 2017) as well as early experiences of distress (Duque-Alarcón et al., 2019), and in schizophrenia (Friston et al., 2016). Evidence for hierarchical layers of processing in social inference and theory of mind was offered from the work of Diaconescu et al. (2017, submitted).

This kind of “amalgamation” of different sources of contributory evidence does not constitute proof of a theory but may be a critical for development as well as refinement of theory (Fletcher et al., 2019; Kao, 2019). This form of evidence can suggest that a theory is plausible rather than confirm it. In turn, plausibility is an important guide to which theories should be investigated further, and which not (Bertolaso and Sterpetti, 2019).

This form of amalgamation of evidence may be unavoidable when faced with theories that are difficult to prove:

“When access to phenomena of interest is incomplete, piecemeal, indirect, or mediated by substantial auxiliary assumptions, it is not always obvious in what manner scientists can justifiably decide how their total evidence comparatively supports hypotheses and informs future research” (Fletcher et al., 2019, p. 3164).

In this case, the challenge is presented by the likelihood that both part objects and objects are encoded in complex multiple areas of the cortex and involve multi-level processes that unfold over time. This makes it more than challenging to isolate specific objects in brain-imaging research. This challenge can be seen more clearly when one tries to locate the part- and dominant-object models in Panksepp’s scheme of emotions, the primary, secondary, and tertiary emotions. At their outset, when part objects (and the beginnings of the dominant model) form, they fit most closely with the secondary layer described by Panksepp (2010), in which they are shaped by basic learning processes not dependent on any tertiary-level processes in the beginning. However, if we try find some consilience between the tertiary-level processes described by Panksepp on the one hand, and the consideration of alternative policies of action that have reached sufficient “temporal thickness” or “counterfactual depth” (which Friston, 2018, described as foundational to consciousness) on the other, we could say that both part-object models and dominant models are reflected in tertiary level processes as well (though the dominant ones usually much more so). Clearly, both must involve some encoding at a cortical level, though with dominant models probably reflected by more connections and distribution than part-object ones.

In this way, cortical representation of long-term memory must play a role in the formation of platonic models. While it has been suggested above that part-object models are more than just memory representations of perceptual experiences, action selection is an inherent aspect of working memory, which activates those representations. In his paper “Cortex and Memory: Emergence of a New Paradigm,” Fuster (2009) describes a situation demonstrating this difficulty with regard to long-term memory networks, which become activated in working memory:

“… [A] memory or an item of knowledge consists of a widespread cortical network of connections, formed by experience, that joins dispersed cell populations. … A complex memory network, … is largely interregional, linking neuron assemblies and smaller networks in separate and noncontiguous areas of the cortex” (p. 2048).

These challenges do not mean that proof is impossible. However, the requirement in this case would require brain imaging data that compare transient states such as in intense emotions conceptually related to part objects, with longitudinally obtained data of brain states in early childhood, to say if they are similar. This is of course made difficult due to the changes that occur in maturation.

In the absence of such evidence, system models of this kind often make use of different strategy that involves simulation and application of mathematical modeling.

“… [W]e start with verbal models that explicate interactions and that in some cases are presented in graphs. Usually the next step should be a mathematical formalization of this hypothetical causal model but we don’t think this will really increase evidence here and therefore it should be reserved for a later step of discussions of modeling the mind. After the formalization, empirical data should be integrated and now it is possible to transform the model to a computer algebra system (e.g., Maple R, Matlab R, Mathematica R) for running simulations in order to explore the functional structure of the model by process analysis. This stepwise procedure was developed basically in the context of systems dynamics ….” (Tretter and Löffler-Stastka, 2018).

The study by Moutoussis et al. (2014b) is an example of such application of a mathematical model applied to a simulation, and the results compared with what is expected. It is hoped that the present work might stimulate further research of a similar kind, which may model the relative influence of part object and dominant models of people.

## Author Contributions

The author confirms being the sole contributor of this work and has approved it for publication.

### Conflict of Interest

The author declares that the research was conducted in the absence of any commercial or financial relationships that could be construed as a potential conflict of interest.

## Footnotes

^1^Strictly, this is incorrect – all perception must be in terms of mental representations, and there is no direct perception of reality. Rather, what is implied are poles of a dimension, that is apparently more reality driven versus representation driven.

^2^This formulation and the equation specifying the free energy principle can be found by Friston (2010).

^3^This refers to Plato’s notion of an ideal form of a thing. For example, one might consider all the persons you have encountered in reality as reflections of a prototypical form of a person, which we might call a “platonic” person. The author thanks Dr Jeremy Holmes, who commented on an earlier version of this paper, and suggested the terms "schematic" or "stereotypical" as alternatives to “platonic” here.

^4^Diaconescu et al. (2017, submitted) presented evidence for a temporal sequence of activation of a proposed hierarchy of levels of cortical function for a social perception task that required different levels of processing of social information. The findings suggested that all areas typically associated with “theory of mind” tasks were activated during the sequence, such as the middle cingulate gyrus, medial prefrontal cortex, and temporo-parietal junction.

^5^The slaving principle in physics (Haken, 1983/2004) proposes that slower “macro” processes entrain faster microprocesses. This concept has been applied to levels of neural architecture (Badcock et al., 2019) and levels of organization of the mind in psychoanalysis (Connolly and van Deventer, 2017; Connolly, 2019). The dominant platonic person model, as a much more complex (and accurate) model involving more complex functional connectivity, must also be a slower, “bigger” process than part-models and can entrain them. The present description obviously parallels Kahneman’s (2011) “fast and slow thinking” processes.

^6^This paper has not focused explicitly on the link between one’s self-representation and our object-representations of others. Clearly this is of some importance as there may be some basis for suggesting that self- and other-representations overlap to some extent; for example, Singer et al. (2004) found that activations while apparently observing significant others receiving a pain stimulus overlap with activations when experiencing that same stimulus, though there may be distinct mechanisms for perceiving self and other (Lamm et al., 2016). Even potentially distinct self- and other-representations may influence one another in person perception. Moutoussis et al. (2014a,b) have offered an active inference model of person perception using a simplified Trust game task, which showed that self-representations in the form of preferences of the sort of person I am influence my goals in interaction as well as how I see the other. In the same way, I may infer what sort of person I am (or will be) from how the interaction progresses. This interaction between self- and other-representation requires further development than given in the present paper.

^7^My thanks to Dr. Pieter Grobbelaar, who suggested that at an early age, the self becomes constituted around either predominantly positive emotions or predominantly negative emotions, which tend to persist through the lifespan. This fits the view being expressed in this paper, in which affective experience is organizing experiences of both the self and the others. Given the adaptive value of positive affect in child development and adaptation, caregivers of very young children who wish to facilitate future positive affect in children should aim to maximize the child’s experiences of pleasure and minimize negative emotional experiences in the earliest stages, as far as that is possible.

^8^The view of consciousness taken in this paper follows the description by Hobson et al. (2014) of the highest level of a hierarchy of organization, which is founded upon counterfactual simulation of future consequences of actions of sufficient depth (Friston, 2018).

^9^Eryilmaz et al. (2011) found changes in functional connectivity impacting on resting states following transient emotion.

^10^In a related way, horror movies depicting demonic characters who are only motivated by extreme sadistic empathy may well be so frightening to people because they activate such early part objects organized around emotions of persecutory anxiety or fear. Freud (1919/1955) offered an idea like this in “The Uncanny,” which refers to “… that class of the frightening which leads back to what is known of old and long familiar.” (p. 220). This description of affectively organized generative part-object models may also offer some new life for Jung’s concept of *archetypal figures* in psychology. It would seem interesting to explore how figures such as the great mother, the child, the devil, and the trickster may be related to early experience organized by corresponding (mixtures of) activation of affective command systems. The unique imagery or thematic nature of our personal part objects is a result of our ongoing experiences (some of which are culturally shared) that have updated these models to a small extent.

^11^My thanks to reviewer, Dr. Karl Friston with constructive assistance with this and other points in the paper.

^12^While the difference between BPD and schizophrenia here is given as involving different levels of functional connectivity, the specific patterns of connectivity implicated in both may be distinct. This distinction may be an important area for future research.

^13^Though not addressed in this paper, states of reduced consciousness (e.g. sleep, intoxication, fatigue and others) must also reduce the functioning of the dominant platonic model, thereby increasing part-object influence on consciousness.

^14^Witt et al. (2017) have shown how BPD has some genetic overlap with Bipolar Disorder, Major Depressive Disorder, and Schizophrenia, including genes implicated in cell adhesion and myelination: “The gene-set analyses yielded significant results for exocytosis. In neuronal synapses, exocytosis is triggered by an influx of calcium and critically underlies synaptic signaling. Dysregulated neuronal signaling and exocytosis are core features of neurodevelopmental psychiatric disorders such as the autism spectrum disorders and intellectual disability” (p. 5). It seems possible that these genetic differences all contribute to limits on functional connectivity in various ways that in turn place limits on the potential complexity of generative models that may regulate affective functioning.

^15^Duque-Alarcón et al. (2019) reported finding atypical brain functional connectivity in BPD patients who have experienced childhood maltreatment.

^16^The author worked for several years in a community-based residence for people suffering from psychotic disorders and gained the impression that the patients’ relationship to their core delusion seemed formally similar to an attachment, due to the great importance given to the delusional content, how it was often invoked when dealing with stressors, the distress experienced when it was threatened by information or insensitive interaction in some way, and the efforts to reject or avoid situations or people who threatened these perceptions. The reason being given in this paper is that that delusional content has become the central foundation of the conscious self in many of these patients.

^17^This may be due to some form of progressive deterioration of the dominant model(s) or perhaps to an increase in demands on the person that exposes an existing fragility of the dominant models. Discussions with male patients often revealed that their first acute episodes happened together with an increased demand for autonomy and reduction of parental care, such as entering the army or going away to study. However, this idea seems more difficult to apply to the typically older onset for female patients.
